# FIBER: enabling flexible retrieval of electronic health records data for clinical predictive modeling

**DOI:** 10.1093/jamiaopen/ooab048

**Published:** 2021-08-02

**Authors:** Suparno Datta, Jan Philipp Sachs, Harry FreitasDa Cruz, Tom Martensen, Philipp Bode, Ariane Morassi Sasso, Benjamin S Glicksberg, Erwin Böttinger

**Affiliations:** 1 Digital Health Center, Hasso Plattner Institute, University of Potsdam, Potsdam, Germany; 2 Hasso Plattner Institute for Digital Health at Mount Sinai, Icahn School of Medicine at Mount Sinai, New York, New York, USA; 3 Department of Genetics and Genomic Sciences, Icahn School of Medicine at Mount Sinai, New York, New York, USA

**Keywords:** databases, factual, electronic health records, information storage and retrieval, workflow, software/instrumentation

## Abstract

**Objectives:**

The development of clinical predictive models hinges upon the availability of comprehensive clinical data. Tapping into such resources requires considerable effort from clinicians, data scientists, and engineers. Specifically, these efforts are focused on data extraction and preprocessing steps required prior to modeling, including complex database queries. A handful of software libraries exist that can reduce this complexity by building upon data standards. However, a gap remains concerning electronic health records (EHRs) stored in star schema clinical data warehouses, an approach often adopted in practice. In this article, we introduce the FlexIBle EHR Retrieval (FIBER) tool: a Python library built on top of a star schema (i2b2) clinical data warehouse that enables flexible generation of modeling-ready cohorts as data frames.

**Materials and Methods:**

FIBER was developed on top of a large-scale star schema EHR database which contains data from 8 million patients and over 120 million encounters. To illustrate FIBER’s capabilities, we present its application by building a heart surgery patient cohort with subsequent prediction of acute kidney injury (AKI) with various machine learning models.

**Results:**

Using FIBER, we were able to build the heart surgery cohort (*n* = 12 061), identify the patients that developed AKI (*n* = 1005), and automatically extract relevant features (*n* = 774). Finally, we trained machine learning models that achieved area under the curve values of up to 0.77 for this exemplary use case.

**Conclusion:**

FIBER is an open-source Python library developed for extracting information from star schema clinical data warehouses and reduces time-to-modeling, helping to streamline the clinical modeling process.

## INTRODUCTION

The advent of large-scale electronic health record (EHR) databases has paved the way for promising applications in precision medicine.^1^^,^^2^ By providing access to retrospective cohorts of real-world patient populations, they constitute a powerful instrument for clinical research.^3^ Furthermore, advancements in machine learning (ML) in the last decade led to a surge of ML algorithms applications on EHR data.^4^ However, both defining the inclusion and exclusion criteria for specific disease cohorts as well as extracting their data from the databases is a sophisticated process.^5^ Translating these workflows into database queries and transforming the extracted data for further analysis tasks requires deep-ranging knowledge of the underlying EHR data model, its entities, and their relationships. Those skills often take a considerable amount of time to develop and lead to code that is hard to maintain and replicate. Enabling researchers to work with the data directly in their modeling pipeline, abstracting away database structures and vendor-specific jargon, promises to reduce time-to-modeling in clinical ML applications.^6^

To that effect, different initiatives related to interoperability have been championed by industry and academia.^6^ Three of the most prominent ones are the Fast Healthcare Interoperability Resources (FHIR),^7^ the Observational Medical Outcomes Partnership (OMOP) Common Data Model (CDM),^8^ and the Integrating Biology and the Bedside (i2b2) clinical data warehouse platform. Only the latter one is inspired by a “star schema” design:^9^^,^^10^ in this schema, atomic “facts”, that is, single observations about a patient, are stored in a narrow fact table and ontology tables are then used to translate local database codes to medical concepts.^11^ As of January 2021, 83 academic health centers in the United States are currently using the i2b2 data model, which builds upon the star schema design.^12^ While high-level programming languages libraries for querying OMOP-based data warehouses are available, no such open-source tools exist for star schema-based EHR systems to our knowledge.^6^^,^^13^^,^^14^ We thus created the FlexIBle EHR Retrieval (FIBER), an open-source Python library which is addressing this gap by providing an end-to-end framework that allows users to efficiently and reliably extract EHR data from star schema-based databases for easy integration into ML pipelines. To illustrate the use of FIBER, we present a concrete use case for building a cohort of heart surgery patients who developed acute kidney injury (AKI). Additionally, we evaluate the query-time performance of the framework in two different databases, one columnar In-Memory Database (IMDB) and another following a traditional row-based approach.

## RELATED WORK

This section describes the cohort building process and the software tools currently available for that intent.

### Cohort building methodology

EHR databases and early standardized data formats were originally designed for billing and accounting purposes, and not primarily intended for conducting scientific medical research.^15^ This secondary use has been a product of a wider and more abundant availability of routine clinical data from Hospital Information Systems (HISs) in such databases. Since most research questions involving EHR databases focus on specific patient subsets, that is, cohorts, this development came along with the need for defining concise criteria to select such cohorts. This process is called phenotyping and a number of initiatives aim at providing such validated sets of criteria to the EHR and medical research community.^5^^,^^16^ A typical phenotyping workflow would comprise a connection to the EHR database, setup of the case-specific vocabulary, querying different data modalities, that is, structured (diagnosis and procedure codes, laboratory values, medications, vital signs, etc.) and unstructured—mostly clinical notes—and combining these modalities in a conditional way to define different subcategories of (diseased) cases and (healthy) controls, for example, in the context of heart disease.^17^ In a star schema data structure, these different events during a patient’s journey can be found in a *fact* table. In order to make sense of these atomic facts, every of these entries needs to be connected to information in other dimension tables such as diagnoses and laboratory values. Additionally, the temporal occurrence of these events often needs to be taken into account. In practice, this translates to complex and nested SQL queries including many *JOIN* operations, which can become computationally expensive. The retrieved cohort data should then be transformed in a format that easily allows for processing and follow-up analysis.

### Software tools for cohort building and analysis

The existing tools in the area of applied EHR-based research are tailored around the creation and assessment of cohorts and their basic statistical analysis in the context of observational research or hospital quality improvement programs.^16^^,^^18^^,^^19^ Most of the available software is based on the OMOP medical database schema or proprietary formats and the majority of the tools do not provide modeling-ready, patient-level aggregated features as output. Neither are all published tools available as open-source software.^18^^,^^19^ Some of the existing tools and libraries for EHR analysis and their features are mentioned in Table 1. The selection was based on the open-source availability of the tools and the independence from the underlying HIS. The table should give an overview of key features (though not exhaustive) of the different tools. Each tool was assessed independently by two of the authors. In case of no consensus, a third author was involved into the discussion. The comparison with FIBER is discussed in the Discussion section.

**Table 1.** Major features of cohort creation and analysis tools

Feature	ATLAS^20^	FIDDLE^21^	inspectOMOP^13^	Leaf ^22^	PLP ^23^	ROMOP ^6^	rEHR ^24^	FIBER
Internal standards								
Standard of underlying database	OMOP	Specialized data set only (MIMIC)	OMOP	i2b2+ OMOP	OMOP	OMOP	CPRD	i2b2
(Programming)interface	GUI	Py	Py	GUI	R	R	R	Py
Data handling								
Complex cohort building	●	◐	◐	●	●	◐	●	●
Modeling-ready dataframes (aggregated at patient level)	◐	●	◐	○	◐	◐	◐	●
Customization of graphical display and results	◐	●	●	◐	◐	○	●	●

*Abbreviations*: Py: Python; GUI: graphical user interface; CPRD: Clinical Practice Research Datalink.

Legend: ●: fully supported; ◐: partially supported; ○: not mentioned.

## METHODOLOGY

In this section, we describe the data on which the library was built and tested on. Then, we show the architecture and the design principles that guided the development of the library. Finally, we describe the technology stack used.

### Data description

FIBER was developed and tested using the deidentified EHR data within the Mount Sinai Data Warehouse (MSDW), which uses a star schema data model. The Mount Sinai health system generates a huge volume of structured, semistructured, and unstructured data as part of its healthcare and clinical operations, which include inpatient, outpatient, and emergency encounters.^25^ In this article, we use the structured data which resides in the MSDW. As of the end of 2018, MSDW contains data from more than 8 million patients with more than 120 million encounters. Each encounter between the patient and the hospital generates one or more facts which are stored in the FACT table of the star schema. The FACT table has around 2.4 billion entries. Every fact is associated with the deidentified Medical Record Number (MRN) of a patient. Dimension tables are used to translate concepts, such as “diagnosis of essential hypertension” to local database codes, such as the International Classification of Diseases (ICD). Some facts of the same category are often grouped together when they happen during the same encounter and under the same context. The grouping tables in the database schema help us map the group facts to individual facts. These groups reduce the number of entries in the fact table but can imply larger *join* operations and result in more complicated SQL queries. Testing FIBER with MSDW has allowed us to show that FIBER can handle high volume of real-world EHR data. The underlying data schema is provided in a database embedded in a Docker container (cf. Supplementary Material for further details).

### Software architecture

**Figure 1.**
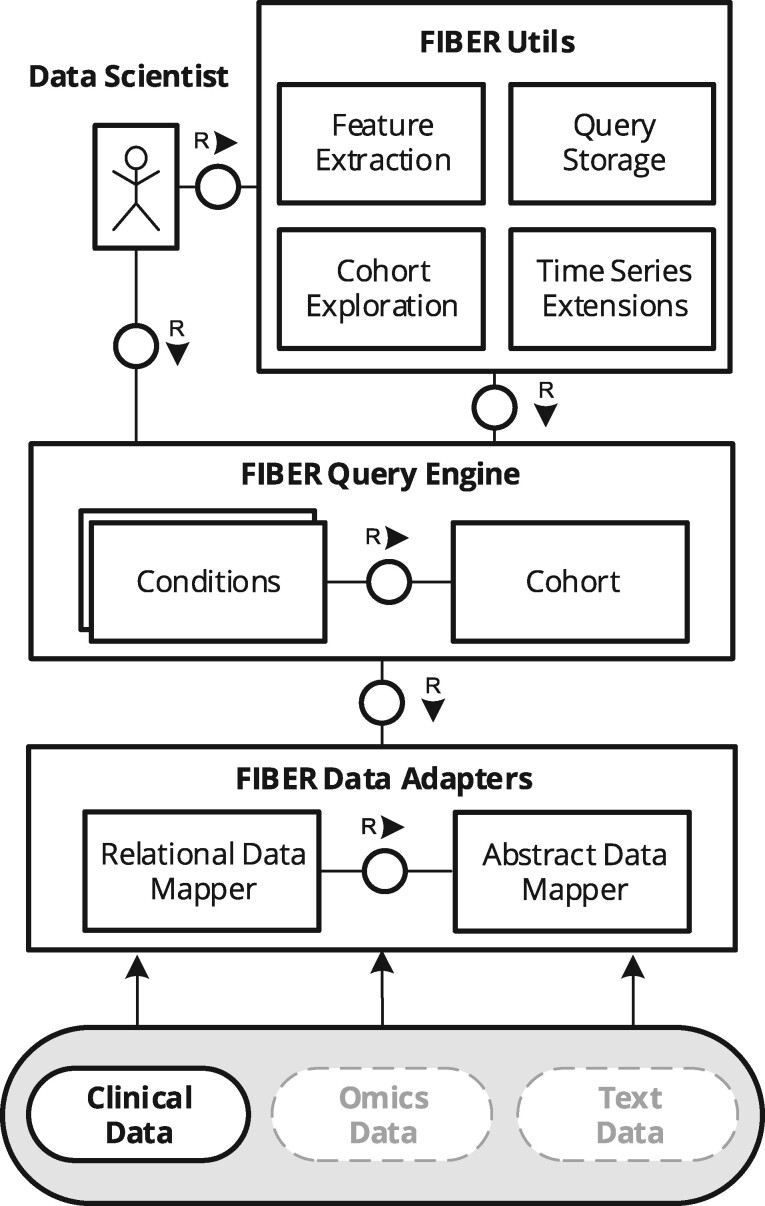
FIBER architecture depicted using a Fundamental Modeling Concepts (FMC) block diagram. The architecture can be extended to Omics and Text Data.

Figure 1 presents the architecture of FIBER with its components using an FMC block diagram.^26^ Clinical data scientists can obtain cohorts and use automated functions through the FIBER Application Programming Interface (API), which is built on top of a unified query engine and data adapters.

#### Data adapters and extensions

FIBER is currently focused on structured clinical data; however, the architecture is flexible to accommodate a number of other data sources. Assuming a suitable data mapper is developed, it becomes possible to tap into not only relational data but also omics or textual data. For instance, a Variant Call Format (VCF) genomic file could be mapped onto a tabular format containing only genetic variants and chromosome positions by a corresponding “VCFDataMapper.” By extending FIBER and creating a “Variant” condition based on this mapper, one could generate cohorts that match a given genomic variant. At this stage, FIBER relies on *SQLAlchemy*^27^ as a mapper for querying relational databases, but the FIBER query engine is agnostic to the underlying data sources. In our example with the heart surgery cohort, the data scientists can specify the conditions which define the case cohort. These conditions are then translated to SQL queries by the FIBER data adapters and are executed against the database to retrieve the patient cohort. In order to achieve reproducibility, the cohort definition can be saved and shared with fellow researchers.

#### Query engine

The query engine holds condition and cohort objects, which are the main building blocks of FIBER. Conditions enable us to filter the FACT table based on different dimensions such as diagnosis, procedure, medications, etc. For example, facts that indicate a heart surgery can be extracted with the following code:

Procedure (description = ‘HEART SURGERY’)

Conditions can return multiple facts about the same patient. For example, if a patient has two heart surgeries, the command above will return two entries for that patient, which are separate in time. Table 2 shows the available condition classes. Apart from textual description supporting partial string matching, standardized coding schemes can also be used to create conditions where available. For example, below are two semantically equivalent procedures which would yield the same result:

**Table 2.** Available condition classes in FIBER

Condition class	Initialization with	
	Description (text)	Clinical codes
Diagnosis	✓	✓
Procedure	✓	✓
Measurement (procedure)	✓	✓
Vital sign (measurement)	✓	✓
Material	✓	✓
Drug (material)	✓	✓
Encounter	✓	–
Metadata	✓	–
Laboratory value	✓	✓^a^
Patient	✓	–

*Note*: Some conditions can only be created from short descriptions, for example, *LabValue(“GLUCOSE”)*, others also from standardized clinical coding scheme like ICD-9, for example, *Procedure(code=*“*35.0*”*, context=* “*ICD-9*”*)*. The condition class names in brackets indicate their parent condition class.

^a^ Standardized codes like LOINC can be integrated into the FIBER framework but have not been applied in the current use case.

Procedure (code=’35.0’, context=’ICD-9’) # OR Procedure (’35.0’,’ICD-9’) Procedure (description=’CLOSED HEART VALVOTOMY’)

Since most cohorts are selected by complex conditions that incorporate more than a single code or group of codes, FIBER allows researchers to combine two or more conditions with Boolean operators AND and OR. Conditions can be combined in any manner and with different nesting levels. The following code, for example, will generate a condition which is satisfied by every patient that is male and has either a closed heart valvotomy (ICD-9 code: “35.0”) or a bypass anastomosis for heart revascularization (ICD-9 code: “36.1”):

(Procedure(’35.0’,’ICD-9’) | Procedure(’36.1’,’ICD-9’)) & Patient(gender=’male’)

The next building block of FIBER is the cohort object. A cohort can be built based on one or more conditions. While a condition describes all occurrences of matching events from the FACT table, a cohort describes the set of patients for which at least one matching occurrence exists. For example, the following code generates a set of patients who had closed heart valvotomy:

Cohort(condition=Procedure(description=‘CLOSED HEART VALVOTOMY’))

Once the cohort has been created, FIBER provides easy utility functions to analyze it further, for example, calculating its size and basic demographic composition. Additionally, two other noteworthy functions are occurs and values_for. The first one allows users to get occurrences of different conditions such as procedures, diagnosis, etc. relative to the cohort in question. The valuesfor function allows researchers to extract different laboratory values and vital signs. The details of all the functions available on the cohort object can be found in the documentation of the library.

#### Cohort storage

Apart from the regular cohort building, FIBER also offers utility functions to facilitate the collaboration between scientists working with EHR. One such function enables the researcher to save complex cohort conditions in the JavaScript object notation (JSON) so it can be easily shared among researchers.^28^

#### Feature extraction

FIBER already provides users with functions like occurs and values_for to extract individual diagnosis, procedures, laboratory, and other values, for a particular cohort. Nonetheless, sometimes the researcher does not know upfront which features from the ones available will show significant predictive power for the condition of interest. The number of all possible features can easily run into the order of hundreds of thousands, if not more. Hence, it is not feasible to extract all possible features individually. To address this issue, FIBER provides a simple function to extract all possible features for a particular cohort, within any specified time window. Also, as we run into features in the range of thousands, most of them become very sparse. A lot of medications, diagnosis codes, and laboratory values have entries only for a very small number of patients. While many imputation algorithms exist to impute features that contain null values, researchers usually choose to drop the features that are very sparse. Therefore, FIBER also allows researchers to define thresholds for the different feature classes (laboratory values, diagnoses, etc.), so only features which are present for at least a certain fraction of the total patients are extracted. Since multiple instances of specific laboratory tests can be encountered for a particular patient, FIBER also allows researchers to aggregate these values either by predefined aggregation functions (eg, min, max, mean, and median) or by using a user-defined aggregation function.

#### Time series extensions

EHR data are intrinsically longitudinal in nature. In some ML approaches, such data cannot be used for modeling without prior processing, for example, extracting the mean value for a series of measurements. However, those types of aggregation functions often implicate loss of potentially predictive information. Advanced techniques exist for preprocessing time series that aim to use all data points, such as Symbolic Aggregate approXimation (SAX).^29^ To account for this, FIBER has an accompanying utility package (*FIBER Utils*) with built-in functions for alternative modes for time-series representations and other cohort aggregation functions. These utilities make experimenting and model prototyping more convenient.

#### Cohort exploration

FIBER provides a number of utilities to quickly explore a given cohort. They encompass, among others, distribution of demographic features, heatmap for the number of patient encounters over time, and summary plot for the availability of features given a certain threshold.

### Technology stack

FIBER was developed as a Python 3.x module. It makes extensive use of the object-relational mapper *SQLAlchemy* for interfacing with the database, thus abstracting away the complexities of database-specific SQL dialects.^27^ Within this framework, data are handled by means of Pandas dataframes.^30^ This presents a 2-fold advantage (1) it allows complex data operations to be performed consistently regardless of the underlying data source and (2) it is widely employed in the ML community as the *de facto* standard for data manipulation along with NumPy.^31^ Graphical functionality is provided by the packages Matplotlib and Seaborn.^32^

When working with large amounts of records as in the MSDW, we expect a difference in performance when using distinct database management systems. As FIBER can be connected to different types of databases, its performance has been evaluated on top of both a *MySQL* and an *SAP HANA* database as representatives for row-oriented and column-based database architectures. For the former, a *MySQL* 5.5 instance was selected and for the latter an *SAP HANA* 2.00 running inside a Docker container. All experiments were executed on a server with 64 cores and circa 1 TB of main memory, with the HANA database being limited to 128 GB memory consumption and no predetermined volatile memory cap for *MySQL*. The SQL queries were generated by FIBER and executed on the aforementioned server. To inspect the behavior of the databases with a different number of results, that is, patients or laboratory values, we added a LIMIT clause to the SQL queries.

## EVALUATION

To evaluate our framework, we demonstrate how a concrete cohort building and subsequent predictive modeling use case can be implemented in FIBER using selected utility functions. Additionally, we present performance benchmarks of the framework using three evaluation queries executed into two distinct database types.

### Use case: heart surgery

To illustrate some of the capabilities of the framework, we address the use case of predicting AKI onset after heart surgery using preoperative parameters such as laboratory values, vital signs, comorbidities, and patient demographics. Renal complications affect up to 30% of the patients following cardiac surgery procedures, such as valvectomy or valve replacement and/or bypass surgery, and are associated with poor patient outcomes.^33^ Let us assume a clinical data scientist or researcher aims to create a prediction model for the likelihood of AKI after heart surgery, based on a number of patient characteristics and longitudinal measurements.

In the code snippet below, we first define the condition of the heart surgery. We then build a cohort around that condition. The has_onset function allows us to check which patients in the cohort developed AKI after the heart surgery. The has_onset function automatically extracts the date of the base condition of the cohort (heart surgery) and only considers the AKI events which happened after the surgery. The outcome of this function becomes our target variable. Finally, the get_pivoted_features function helps us to get all the features which we want to input into the model. Here, we only demonstrate this with a sample configuration to extract the laboratory values for this cohort during a time window of 180 days till the day of the heart surgery. Additionally, at least 50% of all the patients must have that laboratory test result. After fetching the laboratory values the function also aggregates them by calculating the minimum, maximum, and median values as indicated in the PIVOT_CONFIG. In a similar fashion, the diagnoses, drugs, and vital signs, etc., can also be extracted for the said cohort. The code performing the whole task looks as follows:

heart_surgery_condition=(

  Procedure(code=’35.%’, context=’ICD-9’).age(min_age=18) |

  Procedure(code=’36.1%’, context=’ICD-9’).age(min_age=18)

)

heart_surgery_cohort=Cohort(heart_surgery_condition) aki=heart_surgery_cohort.has_onset(

  name="aki",

  condition=Diagnosis(code="584.%", context="ICD-9"),

)

PIVOT_CONFIG= {

  LabValue(): {

    ’timewindow’: [-180, 0],

    ’threshold’:0.5,

    ’pivot_table_kwargs’: {’aggfunc’: {’numeric_value’: [’min’,’median’,’max’]}},

  }

}

input_features=heart_surgery_cohort.get_pivoted_features(PIVOT_CONFIG)

Executing this cohort definition, a total of 12 061 patient records (male: 7456; female: 4605) were identified from the database which contains a total of 8 million patient records. Average age in years is 61.6 for male and 61.8 for female. For a more detailed explanation of the code, including the algorithm, please refer to Supplementary Appendix II.

**Figure 2.**
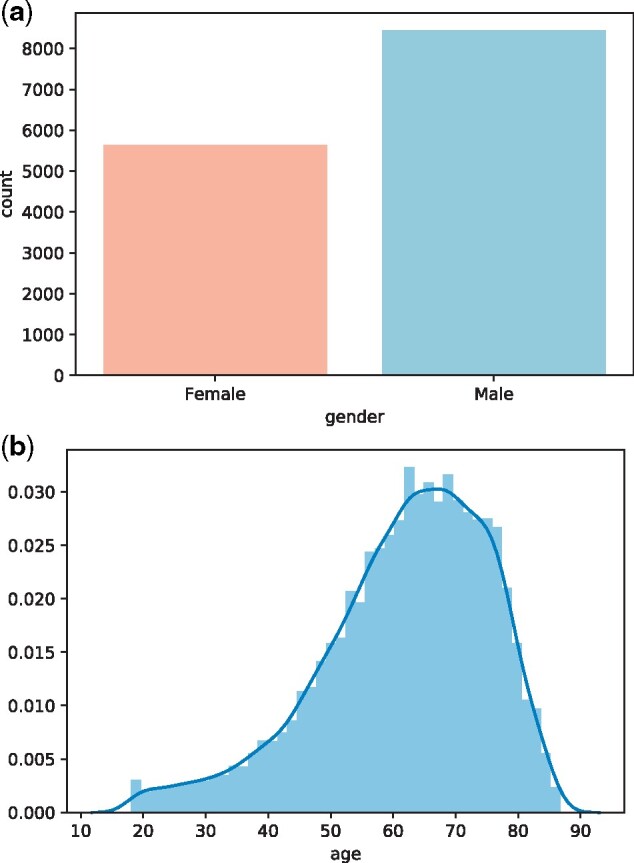
(A) Gender and (B) age distribution plots generated using FIBER for the heart surgery cohort.

Furthermore, we demonstrate a few plotting functions that FIBER provides to explore cohorts. Figure 2A and B show two exemplary plots to inspect the patient demography (age and gender) of the heart surgery cohort that we created above.

**Figure 3.**
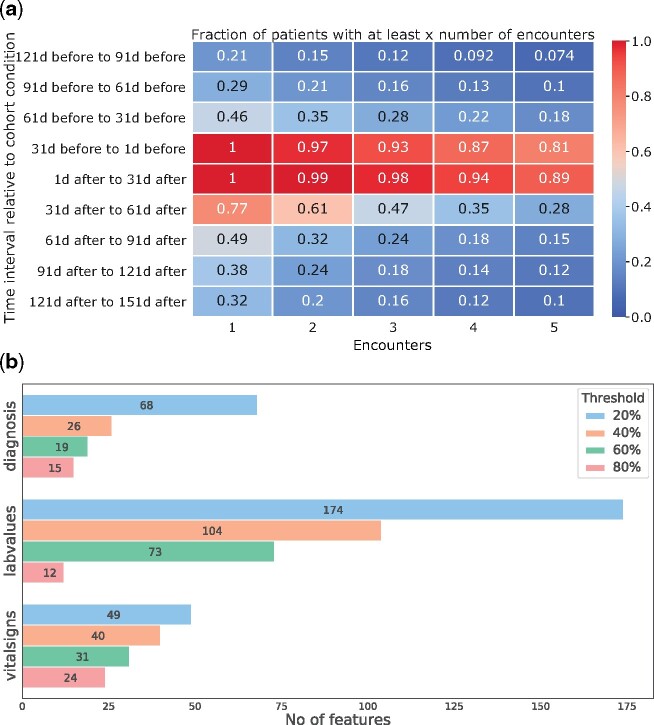
Different utility plots for cohort exploration from FIBER. In (A)—an encounter-timeline plot, the x-axis shows the number of encounters, the y-axis shows different time windows around the heart surgery. The number in the boxes indicates what fractions of patients had that many encounters. In (B)—feature counts, we see the number of features for some of the different feature classes obtained using the get_pivoted_features function for the heart surgery cohort with varying thresholds.

In the use case above, the researcher might also be interested in knowing how many encounters the patients had and how this number changes over time. With data sparsity being a major issue in EHR research, a first glance at data availability for the observation window of interest can be obtained from a built-in function. An example output of the utility function that generates a heat map which shows how the number of encounters of the patients changes around the event of interest can be seen in Figure 3A. This plot shows that the number of patient encounters are higher directly before and after the heart surgery and it gets sparser when moving away from that event. Figure 3B shows the number of features of the different feature classes (diagnosis, laboratory values, etc.) obtained from the get_pivoted_features for the heart surgery cohort for different feature completeness thresholds.

Once the relevant features had been extracted, we predicted the onset of AKI for heart surgery patients. We try two different predictions windows, 7 days and 28 days within which we observe if a heart surgery patient developed AKI or not. We tried four different machine learning models namely logistic regression, random forest, XGBoost, and lightGBM.^34–36^ The models were compared based on the area under the receiver operating characteristics curve (AUC), the area under the precision-recall curve (AUPRC), Precision, and Recall. The mean of these metrics across a 5-fold cross-validation is reported in Table 3. For a more detailed account of the previous work on this specific clinical question and model explanations, please refer to Supplementary Appendix I.

**Table 3.** Metrics for prediction of acute kidney injury onset in a time window of 7 and 28 days after heart surgery, comparing four different models for each prediction period

Prediction window (days)	Model	AUC	AUPRC
7	Logistic regression	0.57	0.10
7	Random_Forest	0.52	0.09
7	Light-GBM	0.73	0.16
7	XGBoost	0.55	0.11
28	Logistic_Regression	0.61	0.18
28	Random_Forest	0.54	0.15
28	Light-GBM	**0.77**	**0.25**
28	XGBoost	0.60	0.20

*Note*: The complete data were extracted with FIBER. The values in bold indicate the best performance achieved.

### Performance benchmarks

For the evaluation, three types of queries that resemble common use cases were executed (1) retrieval of patient MRNs based on a diagnosis, (2) fetching of attributes for those patients, and (3) counting all laboratory results aggregated by type of test. We defined the queries based on the diagnosis codes from the heart surgery use case described in the Use case: heart surgery section.

**Figure 4.**
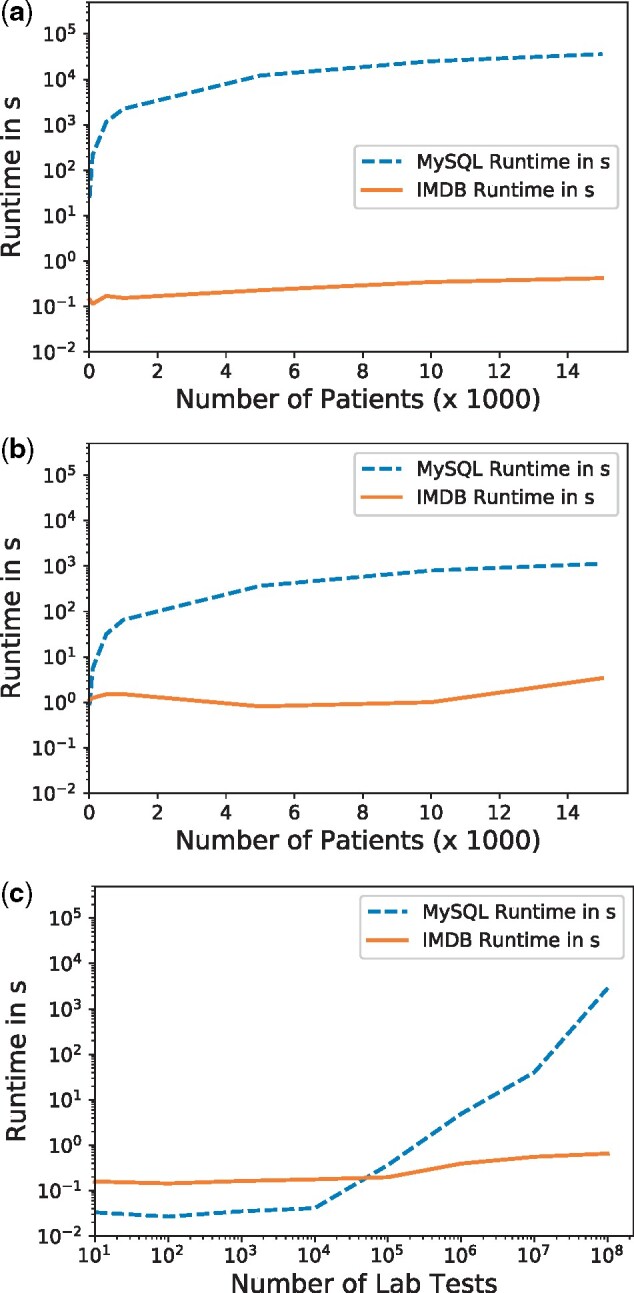
Performance of the FIBER library on two database architectures across three typical use cases. (A) Creation of a cohort with a diagnosis code; (B) fetching of values for a patient cohort; (C) counting lab results by type of test.

In the first query (Figure 4A), the *MySQL* database showed a linear dependency between the number of patients and the runtime. It required nearly 10 h for returning the full result set, whereas the IMDB returned the result set within seconds for all cohort sizes. Moreover, the runtime did not increase as sharply with the size of the result set.

In the second query (Figure 4B), the IMDB outperforms *MySQL*. For the whole cohort, the IMDB returned the values in about 3 s, while the *MySQL* required roughly 20 min and showed the same linear dependency as before. For the third query, we simulated the data exploration feature for laboratory tests by counting the number of patients for different types of glucose tests. In this case, we limited the number of database rows retrieved by using the filter “glucose.” Both databases answered the query within one second for up to 100 000 lab tests, with the *MySQL* database being marginally faster. While the IMDB was still in the subsecond range for the maximum 100 000 000 rows, the *MySQL* database required 45 min for this aggregation and showed an exponential increase for more than 100 000 tests (Figure 4C).

## DISCUSSION

In this section, we address the comparison of FIBER against other libraries, the performance differences observed regarding the usage of different database types, as well as inherent limitations.

### Comparison to state-of-the-art

Here, we shortly discuss the comparison of FIBER to some of the current state-of-the-art libraries and tools for EHR processing that are openly available. The detailed comparison can be found in Table 1 in the Software Tools for Cohort Building and Analysis section. It supports complex phenotyping workflows and enables researchers to conveniently identify cases and controls for the condition of interest. Also, it helps researchers to directly extract machine learning-ready data frames, for example, to get all relevant features for a cohort aggregated on patient level within a specified time window. Noticeably, only one of the tools reviewed directly supported getting all features for a cohort as FIBER does.^21^ Providing a Python interface and working on an i2b2 star schema data format, FIBER stands out in facilitating information exchange and cohort comparability between different health organizations following this schema (eg, the JSON cohort definitions can easily be shared across institutions). Generalizability of data extraction pipelines for these institutions has always been challenging, and we anticipate FIBER to alleviate this issue.

### Performance benchmarks

The differences in runtime observed can be largely attributed to the different database architectures. Patient health information in the MSDW is stored in a star schema format. As such, the benchmark queries join data from the dimension tables to the main FACT table to then filter only the relevant information. In our case, the large volume of the FACT table, which contains 2.4 billion rows, and the necessary *JOIN* operations with other dimension tables makes column-based in-memory databases more performant. In contrast to a row-based database like *MySQL*, columnar databases like the IMDB utilize an optimized memory layout in which a column is represented as a sequence of encoded and compressed values. This architecture reduces the need for I/O during table scans and aggregations such as cohort creation. Since not all columns need to be returned in the query results, this works in favor of the column-based databases. Further, this ability to store sequential values in consecutive memory blocks enables optimization techniques such as data cache prefetching. This difference between row- and column-based databases has been a long-standing topic in database research.^37^ As such, using FIBER makes it possible to write the extraction queries only once which can then take advantage of different database architectures for increased query performance.

### Limitations

Though FIBER enables improvements in current processes of clinical predictive modeling on EHR databases, it does have certain shortcomings. The major limitation is that FIBER is currently only compatible with i2b2 star schema-based databases. As such, in its current state, FIBER does not support out-of-the-box ontology mappings, that is, does not directly interface with standardized ontologies like SNOMED CT.^38^ Future iterations of FIBER may interface with other EHR data models, such as OMOP, which in turn enables ontology mappings to SNOMED CT. Moreover, the library does not address the privacy concerns related to EHR data, for example, anonymization of the underlying data.

## CONCLUSION

In this article, we introduce the Python library FIBER, a novel framework which more seamlessly enables data extraction and modeling for ML on star schema EHR data. This process traditionally requires the stitching of multiple programming languages and packages. FIBER facilitates this process by providing the major functionalities of EHR data extraction and processing in a single, easy-to-use framework. We discuss in detail the functionalities and architecture of the library and demonstrated its capabilities on a real-world clinical predictive modeling use case. Moreover, we show how column-oriented, in-memory databases can also have significant performance gains over row-oriented databases in extracting health data. The code, including detailed documentation and examples, is available as open-source software (cf. Supplementary Material). In future work, the library may be extended to additional health data modalities.

## SUPPLEMENTARY MATERIAL

Supplementary material is available at Journal of the American Medical Informatics Association online.

## AUTHORS’ CONTRIBUTIONS

S.D., J.P.S., H.F.C., and A.M.S. conceptualized the overall study and designed the framework. T.M. and P.B. implemented the framework into a python library. H.F.C. and T.M. carried out the cohort extraction and data cleaning. S.D., J.P.S., and H.F.C. designed the clinical use case, performed the model development and carried out the model performance evaluation. S.D., J.P.S., H.F.C., and A.M.S. drafted the manuscript. B.S.G. and E.B supervised the research project. All authors reviewed the manuscript critically for scientific content, and all authors approved the manuscript.

## Supplementary Material

ooab048_Supplementary_Data

## References

[1] Jensen PB , Jensen LJ, Brunak S. Mining electronic health records: towards better research applications and clinical care. Nat Rev Genet 2012; 13 (6): 395–405. 22549152 10.1038/nrg3208

[2] Glicksberg BS , Johnson KW, Dudley JT. The next generation of precision medicine: observational studies, electronic health records, biobanks and continuous monitoring. Hum Mol Genet 2018; 27 (R1): R56–R62. 29659828 10.1093/hmg/ddy114

[3] De Moor G. EHR-certification, semantic interoperability and the link to clinical research. In: eHealth-WoHIT Conference; 2010; Barcelona, Spain.

[4] Rose S. Machine learning for prediction in electronic health data. JAMA Netw Open 2018; 1 (4): e181404. 30646089 10.1001/jamanetworkopen.2018.1404

[5] Kirby JC , Speltz P, Rasmussen LV, et al PheKB: a catalog and workflow for creating electronic phenotype algorithms for transportability. J Am Med Inform Assoc 2016; 23 (6): 1046–52. 27026615 10.1093/jamia/ocv202 PMC5070514

[6] Glicksberg BS , Oskotsky B, Giangreco N, et al ROMOP: a light-weight R package for interfacing with OMOP-formatted electronic health record data. JAMIA Open 2019; 2 (1): 10–4. 31633087 10.1093/jamiaopen/ooy059 PMC6800657

[7] Bender D , Sartipi K. HL7 FHIR: an Agile and RESTful approach to healthcare information exchange. In: Proceedings of the 26th IEEE International Symposium on Computer-based Medical Systems; IEEE; 2013: 326–31; Maribor, SLOVENIA.

[8] Hripcsak G , Duke JD, Shah NH, et al Observational Health Data Sciences and Informatics (OHDSI): opportunities for observational researchers. Stud Health Technol Inform 2015; 216: 574–8. 26262116 PMC4815923

[9] Murphy SN , Weber G, Mendis M, et al Serving the enterprise and beyond with informatics for integrating biology and the bedside (i2b2). J Am Med Inform Assoc 2010; 17 (2): 124–30. 20190053 10.1136/jamia.2009.000893 PMC3000779

[10] Kimball R , Ross M. The Data Warehouse Toolkit: The Complete Guide to Dimensional Modeling. Hoboken: John Wiley & Sons; 2011.

[11] Klann JG , Abend A, Raghavan VA, Mandl KD, Murphy SN. Data interchange using i2b2. J Am Med Inform Assoc 2016; 23 (5): 909–15. 26911824 10.1093/jamia/ocv188 PMC4997035

[12] Integrating Biology and the Bedside (i2b2). i2b2 installations. https://www.i2b2.org/work/i2b2_installations.html. Accessed January 25, 2020.

[13] Badger J. *InspectOMOP.* https://readthedocs.org/projects/inpsectomop. Accessed March 6, 2020.

[14] Glicksberg BS , Oskotsky B, Thangaraj PM, et al PatientExploreR: an extensible application for dynamic visualization of patient clinical history from electronic health records in the OMOP common data model. Bioinformatics 2019; 35 (21): 4515–8. 31214700 10.1093/bioinformatics/btz409 PMC6821222

[15] Hersh WR. Adding value to the electronic health record through secondary use of data for quality assurance, research, and surveillance. Am J Managed Care 2007; 81 (6 Part 1): 277–8. 17567224

[16] Denaxas S , Gonzalez-Izquierdo A, Direk K, et al UK phenomics platform for developing and validating electronic health record phenotypes: CALIBER. J Am Med Inform Assoc 2019; 26 (12): 1545–59. 31329239 10.1093/jamia/ocz105 PMC6857510

[17] Bielinski SJ , Pathak J, Carrell DS, et al A robust e-epidemiology tool in phenotyping heart failure with differentiation for preserved and reduced ejection fraction: the electronic medical records and genomics (eMERGE) network. J Cardiovasc Transl Res 2015; 8 (8): 475–83. 26195183 10.1007/s12265-015-9644-2 PMC4651838

[18] Tao S , Cui L, Wu X, Zhang GQ; American Medical Informatics Association. Facilitating cohort discovery by enhancing ontology exploration, query management and query sharing for large clinical data repositories. AMIA Annu Symp Proc 2018; 2017: 1685–94. 29854239 PMC5977665

[19] Horvath MM , Rusincovitch SA, Brinson S, Shang HC, Evans S, Ferranti JM. Modular design, application architecture, and usage of a self-service model for enterprise data delivery: the Duke Enterprise Data Unified Content Explorer (DEDUCE). J Biomed Inform 2014; 52: 231–42. 25051403 10.1016/j.jbi.2014.07.006 PMC4335712

[20] Observational Health Data Sciences and Informatics program. OHDSI ATLAS Documentation. https://github.com/OHDSI/ATLAS/wiki, Accessed March 6, 2020.

[21] Tang S , Davarmanesh P, Song Y, Koutra D, Sjoding MW, Wiens J. Democratizing EHR analyses with FIDDLE: a flexible data-driven preprocessing pipeline for structured clinical data. J Am Med Inform Assoc 2020; 27 (12): 1921–34. 33040151 10.1093/jamia/ocaa139 PMC7727385

[22] Dobbins NJ , Spital CH, Black RA, et al Leaf: an open-source, model-agnostic, data-driven web application for cohort discovery and translational biomedical research. J Am Med Inform Assoc 2019; 27 (1): 109–18. 10.1093/jamia/ocz165 PMC6913227 31592524

[23] Reps JM , Schuemie MJ, Suchard MA, Ryan PB, Rijnbeek PR. Design and implementation of a standardized framework to generate and evaluate patient-level prediction models using observational healthcare data. J Am Med Inform Assoc 2018; 25 (8): 969–75. 29718407 10.1093/jamia/ocy032 PMC6077830

[24] Springate DA , Parisi R, Olier I, Reeves D, Kontopantelis E. rEHR: an R package for manipulating and analysing electronic health record data. PLoS One 2017; 12 (2): e0171784. 28231289 10.1371/journal.pone.0171784 PMC5323003

[25] Miotto R , Li L, Kidd BA, Dudley JT. Deep patient: an unsupervised representation to predict the future of patients from the electronic health records. Sci Rep 2016; 6: 26094. 27185194 10.1038/srep26094 PMC4869115

[26] Knopfel A , Grone B, Tabeling P. Fundamental Modeling Concepts: Effective Communication of IT Systems. John Wiley & Sons, Inc.; 2006.

[27] Bayer M. SQLAlchemy. In: Brown A, Wilson G, eds. The Architecture of Open Source Applications Volume II: structure, Scale, and a Few More Fearless Hacks. aosabook.org; 2012.

[28] Bourhis P , Reutter JL, SuáRez F, Vrgocˇ D. JSON: data model, query languages and schema specification. In: Proceedings of the 36th ACM SIGMOD Symposium on Principles of Database Systems. PODS ’17. New York, NY, USA: Association for Computing Machinery; 2017: 123–135.

[29] Lin J , Keogh E, Wei L, Lonardi S. Experiencing SAX: a novel symbolic representation of time series. Data Min Knowl Disc 2007; 15 (2): 107–44.

[30] McKinney W. Pandas: a foundational python library for data analysis and statistics. In: Python for High Performance and Scientific Computing, vol. 14 (9). IEEE; 2011: 1–9; Seattle, WA, USA.

[31] Oliphant TE. A Guide to NumPy, vol. 1. Trelgol Publishing USA; 2006.

[32] Bisong E. Matplotlib and seaborn In: Building Machine Learning and Deep Learning Models on Google Cloud Platform. Berkeley, CA: Apress; 2019: 151–65.

[33] O’Neal JB , Shaw AD, Billings FT. Acute kidney injury following cardiac surgery: current understanding and future directions. Crit Care 2016; 20 (1): 187. 27373799 10.1186/s13054-016-1352-z PMC4931708

[34] Chen T , Guestrin C. Xgboost: a scalable tree boosting system. In: Proceedings of the 22nd ACM SIGKDD International Conference on Knowledge Discovery and Data Mining; New York, NY, USA: Association for Computing Machinery; 2016: 785-94; San Francisco, CA, USA.

[35] Ke G , Meng Q, Finley T, et al LightGBM: a highly efficient gradient boosting decision tree. In: Advances in Neural Information Processing Systems; New York, NY, USA: Curran Associates Inc.; 2017; 3146–54; Long Beach, CA, USA.

[36] Breiman L. Random forests. Mach Learn 2001; 45 (1): 5–32.

[37] Abadi DJ , Madden SR, Hachem N. Column-stores vs. row-stores: how different are they really? In: *Proceedings of the 2008 ACM SIGMOD International Conference on Management of Data*. New York, NY, USA: Association for Computing Machinery; 2008: 967–80; Vancouver, Canada.

[38] Spackman KA , Campbell KE, Côté RA. SNOMED RT: a reference terminology for health care. In: *Proceedings of the AMIA Annual fall symposium*. American Medical Informatics Association; 1997: 640; Bethesda, MD, USA. PMC2233423 9357704

