# Supplementary material for: FIBER: enabling flexible retrieval of electronic health records data for clinical predictive modeling
Source: JAMIA Open. 2021 Aug 2;4(3):ooab048. doi: 10.1093/jamiaopen/ooab048 (PMC8327378; doi:10.1093/jamiaopen/ooab048)
Supplement: ooab048_Supplementary_Data [file ooab048_supplementary_data.zip › APPENDIX I.docx]

**APPENDIX I**

More information about the clinical modeling use case, i.e., prediction of AKI after heart surgery, can be found in this section. Table 4 gives an overview on relevant papers answering the same research question, including cohort sizes, the ML algorithm, and their respective AUROCs.

***Table 4***. Performance of models for AKI following heart surgery.

| **Type** | **Model** | ***N*** | **Model** | **AUROC** |
| --- | --- | --- | --- | --- |
| Regression Models | Cleveland score [1] | 33,217 | LR | 0.81 |
|  | STS score [2] | 86,009 |  | 0.83 |
|  | AKICS score [3] | 603 |  | 0.84 |
|  | SRI score [4] | 2,566 |  | 0.78 |
|  | Ng et al. [5] | 28,422 |  | 0.77 |
|  | Jiang et al. [6] | 7,233 |  | 0.74 |
| Machine Learning Models | Thottakkara et al. [7] | 50,318 | GAM | 0.85 |
|  | Legrand et al. [8] | 212 | SL | 0.76 |
|  | Eyck et al. [9] | 810 | NS | 0.83 |
|  | Kate et al. [10] | 25,521 | EL | 0.74 |
|  | Flechet et al. [11] | 2,123 | RF | 0.84 |
|  | Lee et al. [12] | 2,010 | XGBoost | 0.78 |

Our model was built on a cohort of 12,061 heart surgery patients, out of which 1,005 developed AKI using 774 relevant features. In Figure 4, we show the 10 most important features for the prediction of AKI in our model. This selection is based on those features with the highest mean absolute SHAP (SHapley Additive exPlanations) values, a method to assess feature importance in ML.


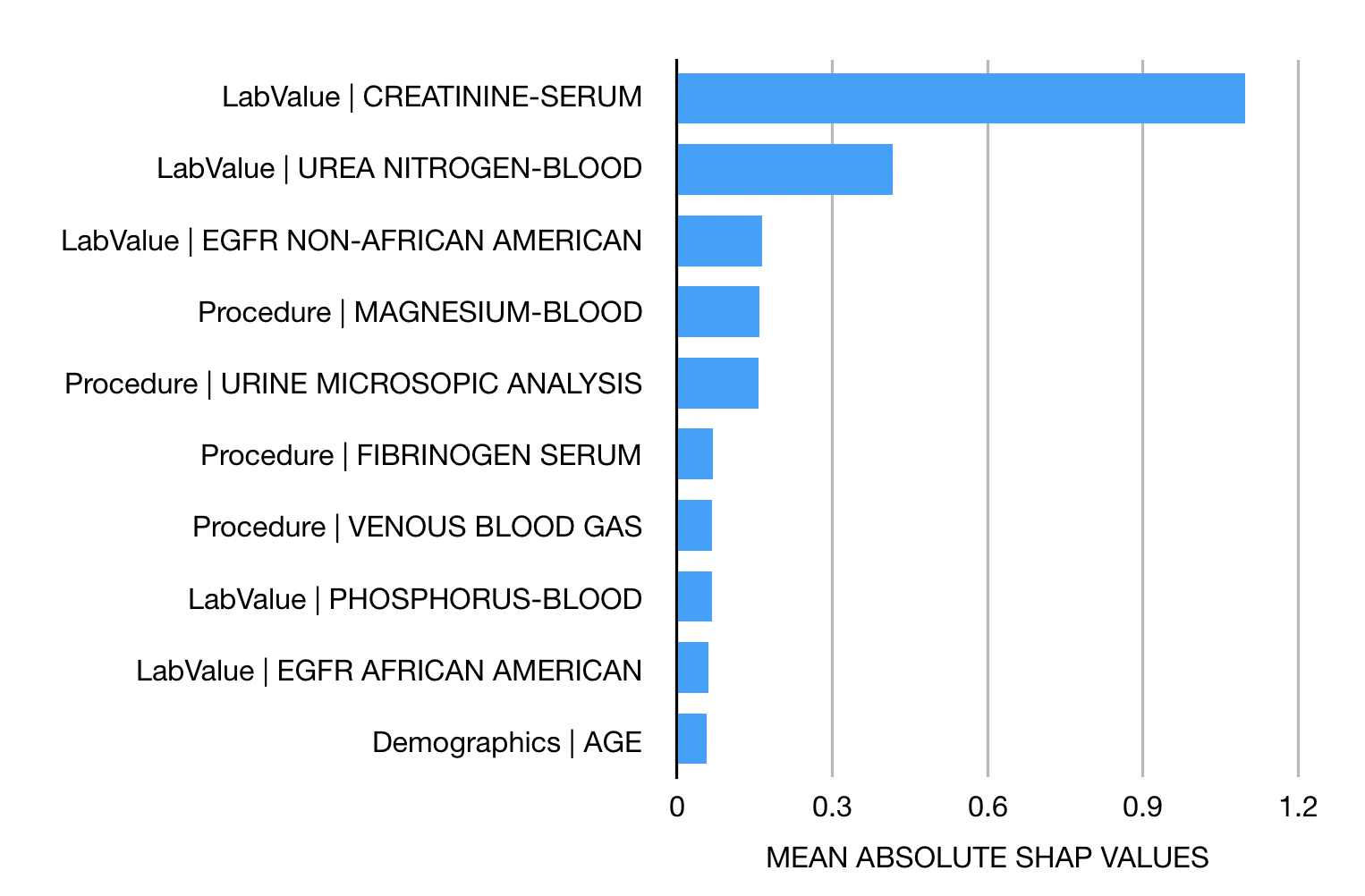


**Figure 5:** Mean absolute SHAP Values for the most important features for predicting AKI for heart surgery patients. The condition class (procedure, lab value etc.) of each feature is also indicated in the label.

1. Charuhas V. Thakar et al. “A Clinical Score to Predict Acute Renal Failure after Cardiac Surgery”. In: J Am Soc Nephrol 14.8 (Aug. 2004)
2. Rajendra H. Mehta et al. “Bedside Tool for Predicting the Risk of Postoperative Dialysis in Patients Undergoing Cardiac Surgery”. In: Circulation 114.21 (2006)
3. H. Palomba et al. “Acute Kidney Injury Prediction following Elective Cardiac Surgery: AKICS Score”. In: Kidney International 72.5 (Sept. 2007)
4. Duminda N. Wijeysundera et al. “Derivation and Validation of a Simplified Predictive Index for Renal Replacement Therapy After Cardiac Surgery”. In: JAMA 297.16 (Apr. 2007)
5. Shu Yi Ng et al. “Prediction of Acute Kidney Injury within 30 Days of Cardiac Surgery”. In: J Thorac Cardiovasc Surg 147.6 (June 2014)
6. Wuhua Jiang et al. “Validation of Four Prediction Scores for Cardiac Surgery-Associated Acute Kidney Injury in Chinese Patients”. In: Braz J Cardiovasc Surg 32.6 (2017)
7. Paul Thottakkara et al. “Application of Machine Learning Techniques to High-Dimensional Clinical Data to Forecast Postoperative Complications”. In: PLoS ONE 11.5 (2016)
8. Matthieu Legrand et al. “Incidence, Risk Factors and Prediction of Post-operative Acute Kidney Injury Following Cardiac Surgery for Active Inefective Endocarditis: an Observational Study.” In: Crit. Care 17.5 (Jan. 2013)
9. J Van Eyck et al. “Data Mining Techniques for Predicting Acute Kidney Injury after Elective Cardiac Surgery”. In: Crit. Care 16.Suppl 1 (2012)
10. Rohit J. Kate et al. “Prediction and Detection Models for Acute Kidney Injury in Hospitalized older Adults”. In: BMC Med. Inform. Decis. Mak. 16.1 (2016)
11. Marine Flechet et al. “AKIpredictor, an on-line Prognostic Calculator for Acute Kidney Injury in Adult Critically Ill Patients”. In: Intensive Care Medicine 43.6 (2017)
12. Hyung-Chul Lee et al. “Derivation and Validation of Machine Learning Approaches to Predict Acute Kidney Injury after Cardiac Surgery”. In: J. Clin. Med. 7.10 (2018).
