# Supplementary material for: FIBER: enabling flexible retrieval of electronic health records data for clinical predictive modeling
Source: JAMIA Open. 2021 Aug 2;4(3):ooab048. doi: 10.1093/jamiaopen/ooab048 (PMC8327378; doi:10.1093/jamiaopen/ooab048)
Supplement: ooab048_Supplementary_Data [file ooab048_supplementary_data.zip › APPENDIX II.docx]

**APPENDIX II**

In this appendix section we explain the code block presented in section 4.1 of the main paper. In the first step we define the procedure conditions(s), i.e., we specify which procedure codes indicate a heart surgery and optionally we can also give a minimum and maximum age of the cohort. In the second step we initialize the cohort object with this condition. This yields a cohort of patients who have undergone heart surgery. Next, we define the target variable, which in this case is achieved by initializing a diagnosis condition with the diagnosis code of AKI. Then we search for the patients in the heart surgery cohort who had an onset of AKI with the *has_onset* function. Finally, we need to extract the features which will be used for the ML model. We do this by first defining the pivot configuration. The pivot configuration tells the algorithm which aggregations to use for a particular condition class, e.g., how much sparsity to allow for. This is particularly useful because EHR data is made up heterogeneous features, some of which are numerical (like lab values), some of them are binary (like diagnosis) and some of them can be categorical (like ethnicity). Hence different aggregation strategies are required for the different data types. Once the pivot configurations are defined, we call *get_pivoted_features* function to automatically extract all the features aggregated according the specification given and potentially filtered by the amount of sparsity allowed. The resulting algorithm for the heart surgery cohort derivation is described below:

| **Algorithm 1.** Obtain heart surgery cohort with pivoted (or aggregated) features |
| --- |
| **Input**: procedure code, age, diagnosis code, time windows, aggregations  **Output**: data frame with features |
| **1** Initialize *procedure condition* with *procedure code*(s) and *minimum age*  **2** Initialize *heart surgery cohort* with *condition*  **3** Initialize *diagnosis condition* with *diagnosis code*  **4** Filter *heart surgery cohort* by *diagnosis code* to set target variable with *has_onset*  **5** Initialize *pivot configuration* with *feature types* and *aggregation types*  **6** Get aggregated features from *heart surgery cohort* using configuration with *get_pivoted_features* |
